# Elucidating effects of environmental exposure using human‐induced pluripotent stem cell disease modeling

**DOI:** 10.15252/emmm.202013260

**Published:** 2022-10-26

**Authors:** Mark Chandy, Detlef Obal, Joseph C Wu

**Affiliations:** ^1^ Stanford Cardiovascular Institute Stanford University School of Medicine Stanford CA USA; ^2^ Department of Medicine Western University London ON Canada; ^3^ Department of Physiology and Pharmacology Western University London ON Canada; ^4^ Department of Anesthesiology, Perioperative, and Pain Medicine Stanford University Stanford CA USA; ^5^ Department of Medicine, Division of Cardiovascular Medicine Stanford University School of Medicine Stanford CA USA

**Keywords:** cannabis, e‐cigarettes, environment, iPSC, opioids, Evolution & Ecology, Methods & Resources, Stem Cells & Regenerative Medicine

## Abstract

Induced pluripotent stem cells (iPSCs) are a powerful modeling system for medical discovery and translational research. To date, most studies have focused on the potential for iPSCs for regenerative medicine, drug discovery, and disease modeling. However, iPSCs are also a powerful modeling system to investigate the effects of environmental exposure on the cardiovascular system. With the emergence of e‐cigarettes, air pollution, marijuana use, opioids, and microplastics as novel cardiovascular risk factors, iPSCs have the potential for elucidating the effects of these toxins on the body using conventional two‐dimensional (2D) arrays and more advanced tissue engineering approaches with organoid and other three‐dimensional (3D) models. The effects of these environmental factors may be enhanced by genetic polymorphisms that make some individuals more susceptible to the effects of toxins. iPSC disease modeling may reveal important gene–environment interactions that exacerbate cardiovascular disease and predispose some individuals to adverse outcomes. Thus, iPSCs and gene‐editing techniques could play a pivotal role in elucidating the mechanisms of gene–environment interactions and understanding individual variability in susceptibility to environmental effects.

## Graphical abstract

In this review, J Wu and colleagues discuss the potential of human iPSC as a modeling system to investigate the toxic effects of environmental exposure on human health.

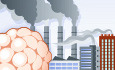

**Glossary**
AHA: American Heart Association
BDNF: Brain‐derived neurotrophic factor
CB1: Cannabinoid receptor 1
CB2: Cannabinoid receptor 2
CRISPR/Cas9: Clustered Regulatory Interspaced Short Palindromic Repeats/Cas9 system
CRISPRi: Clustered Regulatory Interspaced Short Palindromic Repeats Interference
CRISPRa: Clustered Regulatory Interspaced Short Palindromic Repeats Activation
CV: Cardiovascular
DCM: Dilated cardiomyopathy
EGFR: Epithelial growth factor receptor
EHT: Engineered heart tissue
ENDS: Electronic nicotine delivery systems
EVALI: e‐cigarette or vaping use associated lung injury
FDA: Food and Drug Administration
GABA: γ‐aminobutyric‐acid
GDNF: Glial cell‐derived neurotrophic factor
GPCR: G‐protein coupled receptor
HCM: Hypertrophic cardiomyopathy
hERG: Human Ether‐à‐go‐go‐Related Gene
iKR: Inward‐rectifier potassium channels
iPSC: Induced pluripotent stem cell
iPSC‐CM: iPSC‐derived cardiomyocyte
iPSC‐EC: iPSC‐derived endothelial cell
iPSC‐NC: iPSC‐derived neuronal cell
iPSC‐SN: iPSC‐derived sensory neuron
HTS: High‐throughput screening
KLF4: Krüppel‐like factor 4
MEA: Multi‐electrode array
c‐MYC: Myc proto‐oncogene protein
OSKM: Oct2/4, Sox2, Klf4, c‐Myc
PBMC: Peripheral blood mononuclear cells
PDCD4: Programmed cell death protein 4
PGC‐1: Peroxisome proliferator‐activated receptor γ‐coactivator‐1
SCD: Sudden cardiac death
scRNA‐seq: Single‐cell RNA sequencing
SRY: sex determining region Y
SOX2 TALEN: sex determining region Y box 2
TRP: Transcription Activator‐Like Effector Nucleases
Δ^9^‐THC: Transient receptor potential channel delta‐9‐tetrahydrocannabinol

## Introduction

**Figure 1:**
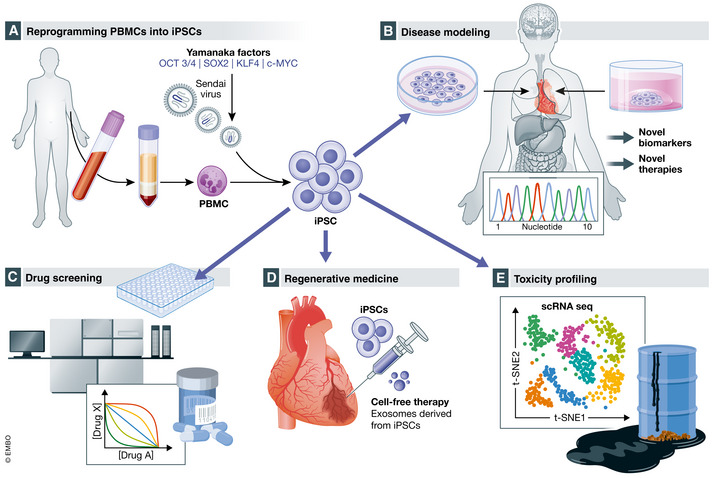
Applications for induced pluripotent stem cells (iPSCs) include disease modeling, drug screening, regenerative medicine, and toxicity profiling (A) From a single blood draw, peripheral blood mononuclear cells (PBMCs) are isolated and reprogrammed into iPSCs via viral transduction and overexpression of the Yamanaka factors (octamer‐binding protein 3/4 (OCT 3/4; also known as POU5F1), SRY (sex determining region Y)‐box 2 (SOX2), Krüppel‐like factor 4 (KLF4), and Myc proto‐oncogene protein (c‐MYC); Takahashi & Yamanaka, 2006; Takahashi *et al*, 2007; Yu *et al*, 2007). (B) iPSCs contain an individual's genetic information, and disease modeling offers the unique opportunity to understand patient‐specific disease mechanisms that might lead to novel biomarkers and therapies. (C) Drug screening traditionally has difficulty translating from small animal and cell culture models to the clinic. iPSCs contain the genetic code of individuals and can be differentiated into any cell type allowing the determination of safety, efficacy, and possibly patient‐specific responses in a dish. (D) Regenerative medicine involves iPSCs and cell‐free therapy with exosomes derived from iPSCs. (E) iPSCs are also ideally suited to testing for the effects of toxins on the different tissue beds and identifying patient‐specific factors that predispose to toxicity. [Colour figure can be viewed at wileyonlinelibrary.com]

The discovery of induced pluripotent stem cells (iPSCs) has transformed the field of stem cell biology and regenerative medicine (Yu *et al*, 2007). Induced pluripotent stem cells (iPSCs) and embryonic stem cells (ESCs) are both pluripotent stem cells (PSCs) capable of self‐renewal and differentiation into any tissue lineage. Tissues derived from ESCs and iPSCs are largely similar from molecular and functional perspectives (Zhao *et al*, 2017). However, the use of iPSCs avoids the ethical problems associated with using ESCs, and iPSCs can be obtained readily from a blood sample to isolate peripheral blood mononuclear cells (PBMCs) or tissue samples with fibroblasts. Adult human somatic cells are reprogramed into stem cells via transferring somatic nuclear material into oocytes, followed by cell fusion, and genetic integration of somatic cell chromatin. The development of viral transduction and overexpression of the Yamanaka factors (octamer‐binding protein 3/4 (OCT 3/4; also known as POU5F1), SRY (sex‐determining region Y)‐box 2 (SOX2), Krüppel‐like factor 4 (KLF4), and Myc proto‐oncogene protein (c‐MYC)) was a revolutionary innovation (Takahashi & Yamanaka, 2006; Takahashi *et al*, 2007; Yu *et al*, 2007). iPSCs can be differentiated into any tissue cell type using a cocktail of recombinant protein factors and small‐molecule inhibitors, allowing the use of cardiovascular tissue for regenerative medicine, disease modeling, and drug discovery (Obal & Wu, 2020; Paik *et al*, 2020). While the financial cost and time required for reprogramming, generating, biobanking, and differentiation are high, iPSCs are a more accurate model of human disease than traditional mammalian cell culture and animal models. Leveraging individual genetic information and recent advances in gene editing (Nishiga *et al*, 2022), iPSCs are proving critical to understanding the molecular mechanisms of human disease and advancing the goals of precision medicine. In addition, with the diverse genetic backgrounds of individuals, a large cohort of iPSC lines has the potential to capture the heterogeneity of disease and drug treatments, which can help us discover the toxic effects of drugs and environmental exposures (Fig 1).

## Regenerative medicine

Despite great advances in percutaneous coronary intervention and medical management, cardiovascular disease remains the world's leading cause of death, with associated long‐term complications that include malignant arrhythmias and pump failure. iPSCs are a limitless source of tissue that are autologous, and their use helps avoid the need for immunosuppression (Lu *et al*, 2013). Despite their initial promise and great enthusiasm, preclinical studies have shown varying and sometimes disparate results for improving cardiac function, vasculogenesis, and reducing apoptosis (Nelson *et al*, 2009; Kawamura *et al*, 2012; Templin *et al*, 2012). Although clinical results from adult stem cells have been mixed, there are now several trials focusing on using human ESC‐ or iPSC‐based cardiovascular therapies (Neofytou *et al*, 2015).

## Drug and toxicity screening

Because they contain an individual's unique genetic makeup, iPSCs are proving to be a powerful platform to discern the beneficial and adverse effects of drugs. Historically, animal models and cell culture systems have provided preclinical safety data for new drugs, and treatments and clinical trials are used to determine safety and efficacy in large heterogeneous populations. While sufficient for most individuals, this approach does not capture the precise cost‐to‐benefit ratio for each person. Currently, iPSCs are expensive and require months to generate. However, once created, iPSCs can be differentiated into any tissue type and provide a limitless supply of tissue for drug and toxicity testing. For example, iPSC‐cardiomyocytes (iPSC‐CMs) can be used to investigate the effects of new antiarrhythmic drugs or caffeine (Luo *et al*, 2021). iPSC‐derived tissue can advance personalized medicine because they are derived from a single individual, making it feasible to predict which treatments are safe and effective for the individual with an enormous potential for achieving the goals of personalized medicine (Lau *et al*, 2019).

## Disease modeling

Traditional methods of studying human diseases using animal models or patient‐derived tissue samples have been valuable, but many findings nevertheless do not translate to the clinic. Animal models do not always faithfully recapitulate the physiology of human disease, and because of interspecies variation, discoveries based on animal findings may not reflect the human pathophysiology (Matsa & Denning, 2012). Patient‐derived tissue is difficult to acquire and limited in quantity, and lacks longevity in cell culture (Beqqali *et al*, 2009). Immortalized cell culture models are complicated by genes that facilitate long‐term culture, and may also disrupt the transcriptome and cell function without reflecting patient‐specific genetic information. Derived from terminally differentiated cells, iPSCs provide an exciting new model that has the potential to transform basic science and precision medicine.

iPSCs can be differentiated into any tissue type, including cardiomyocytes, smooth muscle cells, endothelial cells, and fibroblasts. They can be used to identify genes responsible for a disease or be modified by environmental factors in a dish. Gene‐editing tools, such as TALENS, CRISPR‐Cas9, CRISPR‐I, and CRISPR‐A, can facilitate the discovery of molecular mechanisms of a disease (Hsu *et al*, 2014; Karakikes *et al*, 2017; Ma *et al*, 2018; Nishiga *et al*, 2022). By uncovering novel disease mechanisms, iPSC‐derived tissue is expected to identify novel disease‐specific biomarkers and druggable targets for therapies which may eventually translate into new clinical tools for diagnosis and treatment. Novel disease‐specific biomarkers have the potential to expedite the diagnosis of diseases and facilitate disease management by monitoring response to therapy. iPSCs are also a powerful tool for mechanistic studies that can identify novel drug targets, which are needed to change the scope and dimension of cardiovascular care (Fig 1).

Patient‐specific iPSCs are a limitless source of cardiovascular tissue that have been instrumental in breakthrough studies on the mechanisms of cardiovascular disease such as dilated cardiomyopathy (DCM; Sun *et al*, 2012), hypertrophic cardiomyopathy (HCM; Lan *et al*, 2013), arrhythmogenic right ventricular cardiomyopathy (ARVC; Kim *et al*, 2013; Asimaki *et al*, 2014), left ventricular non‐compaction (LVNC; Kodo *et al*, 2016), and LEOPARD syndrome (Carvajal‐Vergara *et al*, 2010). Imbued with an individual's genetic information, iPSCs are ideally suited for precision medicine (Grskovic *et al*, 2011) and will usher in a new era of biomarkers and therapies for cardiovascular disease. Simultaneously, iPSC‐derived tissues are an excellent platform to evaluate the effects of drugs, toxins, and environmental exposures (Sayed *et al*, 2016; Lee *et al*, 2019). Advances in tissue engineering have allowed for the use of more complex iPSC models such as organoids or engineered heart tissue, which are expected to elucidate interactions between different tissues and cells that contribute to disease pathophysiology (Kim *et al*, 2022).

Besides these applications, a central question is whether iPSCs can be used to study the impact of the environment on cardiovascular disease in this model. Murine embryonic stem cells have been used to study the effects of environmental toxins (Czyz *et al*, 2004a; Czyz *et al*, 2004b; Nikolova *et al*, 2005). The present review examines the advantages and challenges associated with the use of IPSCs in environmental cardiology.

## Environmental cardiology

**Figure 2:**
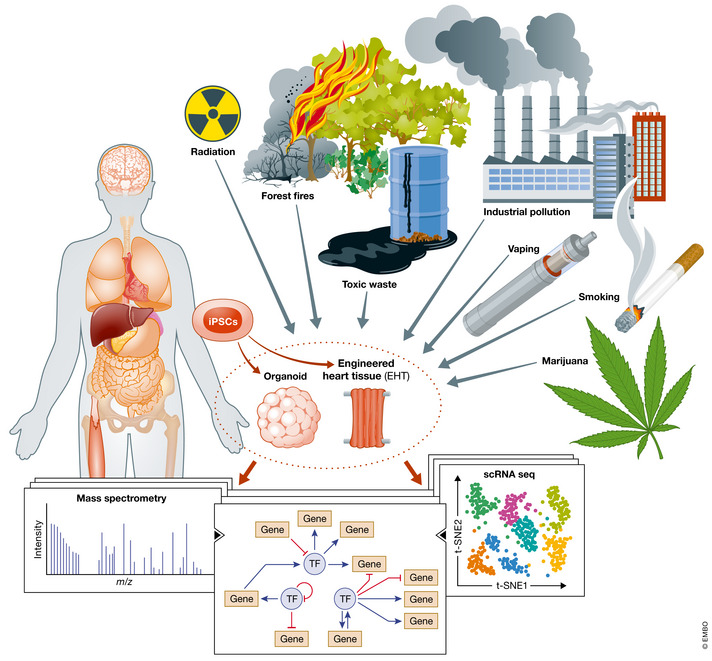
iPSCs offer a unique patient‐specific model to study the effect of the environment on the human body New or unrecognized environmental toxins such as particulate matter 2.5 (PM_2.5_) from forest fires and industrial pollution, marijuana, e‐cigarette, radiation, or toxic waste exposure might cause adverse effects on the human body. iPSC disease modeling is an opportunity to study the effects of environmental toxins before they result in end‐organ damage or organ failure. iPSCs can be differentiated into organ‐specific cells and assembled into fabricated tissues such as engineered heart tissue or differentiated into organoids comprised of organ‐specific tissue. Using single‐cell RNA sequencing (scRNA‐seq), mass spectrometry, and other ‐omic analysis, iPSC disease modeling could be leveraged to discover harmful effects of environmental factors before epidemiology studies are available and prevent significant morbidity and mortality. The discovery of druggable targets and pathways might lead to the development of new biomarkers and therapies for disease. [Colour figure can be viewed at wileyonlinelibrary.com]

The environment is emerging as a significant risk factor for cardiovascular disease (Bhatnagar, 2017). However, the effects of environmental toxins from natural or man‐made sources remain unclear especially in the long term. Despite attempts to curb global warming, industrial pollution, and encroachment into natural habitats, humans are continuously exposed to environmental toxins such as particulate matter less than 2.5 micrometers in size (PM_2.5_; Rajagopalan *et al*, 2020). After years of declining tobacco use, the rise of e‐cigarettes threatens to renew cardiopulmonary disease. The legalization of marijuana has made cannabinoids more accessible around the world, but the long‐term effects of marijuana on the cardiovascular system remain unclear. A recent study revealed that the psychoactive component of marijuana delta9‐tetrahydrocannabinol (Δ^9^‐THC) causes vascular inflammation, oxidative stress in iPSC‐derived endothelial cells, and atherosclerosis in mouse models (Wei *et al*, 2022). Once mislabeled as being non‐addictive, the consequences of chronic marijuana and opioid consumption are now manifesting in adverse cardiovascular outcomes (Jalali *et al*, 2021; Rohani *et al*, 2021). The haphazard use of opioids revealed that a class of medications thought to be powerful tools in preventing pain rapidly became a new environmental hazard affecting some 4% of the U.S. population (Skolnick, 2018). Human iPSCs are a powerful model system to study the toxic effects of these different compounds on the human body (Fig 2).

## Air pollution

The link between air pollution and cardiovascular disease has been described in epidemiological research (Brook *et al*, 2004; Kim *et al*, 2020a; Kim *et al*, 2020b; Kim *et al*, 2020c). About 91% of the world's population inhabits areas with poor air quality that exceeds the World Health Organization (WHO) safety limits, and air pollution is estimated to contribute to over 7 million deaths per year, or 1 in 9 deaths worldwide (Seaton *et al*, 1995; Stieb *et al*, 2002; Venkatesan, 2016). Fossil fuels are an abundant source of not only particulate matter but also carbon dioxide. Climate change mediated by carbon dioxide can lead to drought and wildfires that also exacerbate human exposure to particulate matter. The mega‐fire event experienced in California culminated in a giga‐fire in the fall of 2020 and led to vast amounts of particulate matter transforming the sky into a Mars‐like landscape, a spectacle that is now repeated worldwide. The long‐term effects of this exposure might not be evident in epidemiological studies for decades.

PM_2.5_ are capable of traversing through the lungs and entering the circulatory system. Thus, PM_2.5_ is thought to have adverse effects on the cardiovascular system. Studies in animal models and cell culture suggest that PM_2.5_ can enter the circulation and has the potential to cause adverse effects on the cardiovascular system (Hoek *et al*, 2013; Feng *et al*, 2016). iPSC disease modeling can be used to uncover the toxicities of particulate matter on the body and elucidate the mechanisms of their effects. After exposure to PM_2.5_, iPSC‐CMs were found to have increased arrhythmias due to upregulation of TRM3 (Cai *et al*, 2020). This may account for the increased incidence of sudden cardiac death associated with wildfires (Jones *et al*, 2020). Using iPSC‐derived tissue, investigators may be able to assess the effects of particulate matter on different tissues. The discoveries may inform policymakers and regulatory agencies to craft decisions and laws limiting exposure to PM_2.5_. The continuing advances in iPSC‐based drug discovery promise to an era of novel therapies for cardiovascular disease.

## E‐cigarettes

Smoking has long been associated with lung disease and cancer, and is also a major cause of the cardiovascular disease (CVD), the leading cause of death worldwide (Ambrose & Barua, 2004; Munzel *et al*, 2020). Despite its deleterious effects, tobacco is consumed by 933 million people worldwide (Collaborators, 2017). While tobacco use in developed nations has dramatically improved over the past 50 years, new tobacco products, such as e‐cigarettes, have created a new generation of tobacco users. Since its introduction in 2007, e‐cigarette use among high school students in the U.S. has proliferated, with a more than 900% increase from 2011 to 2018. The latest National Health Interview Survey (NHIS) estimates that 5.5 million (3.8%) US adults were using e‐cigarettes in 2014. Strikingly, over 26.5% of high school students and 15.2% of middle school students were e‐cigarette users (Wang *et al*, 2021).

The toxicity and mechanisms of traditional cigarette smoke on the vascular system are well documented, but the effects of e‐cigarettes on the cardiovascular system have not been systematically studied thus far (Ambrose & Barua, 2004; Johnson *et al*, 2010; Polosa & Caponnetto, 2016; Benowitz & Fraiman, 2017). E‐cigarettes are liquid solutions containing propylene glycol, vegetable glycerin, nicotine, tetrahydrocannabinol (THC), vitamin E acetate, and other components that are vaporized by heating and inhaled as an aerosol. As e‐cigarettes do not involve combustion, they do not produce carbon monoxide or some of the other toxins associated with traditional cigarettes. Initially marketed as a smoking cessation aid, first‐generation e‐cigarettes had a low dose of nicotine and fewer adverse effects, but now e‐cigarette and electronic nicotine delivery systems (ENDS) have evolved to have much higher doses of nicotine. For example, a single pod of Juul™ has the equivalent of 20 cigarettes (one pack) worth of nicotine (Wu *et al*, 2019). The debate about whether e‐cigarettes will provide long‐term benefit or harm is ongoing, with little data on the cardiac and pulmonary effects (Drummond & Upson, 2014). The emergence of e‐cigarette or vaping use‐associated lung injury (EVALI) has further uncovered the adverse pulmonary effects of e‐cigarettes.

Epidemiology studies will likely take years to fully expose the potential adverse effects of e‐cigarettes on the cardiopulmonary system, and in the meantime, e‐cigarette use has grown dramatically without rigorous toxicology assessment. While studies in small animals and *in vitro* cell culture suggested adverse effects of e‐cigarettes on the cardiopulmonary system, iPSC disease modeling was the first to identify that e‐cigarette flavorants had deleterious effects on iPSC‐derived endothelial cells *in vitro* (Lee *et al*, 2019), helping to steer policymakers in the Food and Drug Administration (FDA) to recommend a ban on flavorings.

The effects of e‐cigarettes on embryonic development remain unclear. iPSC‐derived embryoid bodies contain immature cells and tissues that are an ideal platform to screen for environmental toxicities. When combined with single‐cell sequencing, embryoid bodies revealed potentially toxic effects of nicotine on embryonic development (Guo *et al*, 2019). A more rigorous and systematic analysis of the effects of e‐cigarettes on the development is needed.

E‐cigarettes include many hazardous and potentially hazardous compounds affecting the cardiopulmonary system. Nicotine has known adverse cardiovascular effects. Even without combustion, these compounds are toxic but need to be evaluated with a high‐throughput model that provides information on the toxicology of these compounds on human tissue. Genetic polymorphisms contribute to the development of vascular disease with traditional cigarette smoking. Investigators from the CARDIoGRAMplusC4D meta‐analysis found that genetic polymorphisms make certain individuals more susceptible to cardiovascular events (van der Harst & Verweij, 2018; Larsson *et al*, 2020). iPSC disease modeling and transcriptomics can be used to uncover single‐nucleotide polymorphisms (SNPs) that exacerbate cardiovascular disease in the setting of e‐cigarette exposure (Hindy *et al*, 2018; Levin *et al*, 2021). In addition to vascular disease, iPSC disease modeling may provide insights into gene and environment interactions with e‐cigarette components that cause arrhythmias. Toxicity studies in iPSC‐CMs have revealed that cinnamaldehyde is associated with cardiotoxicity and altered cardiomyocyte excitability (Nystoriak *et al*, 2019). More recently, smoking and e‐cigarette exposures were linked to ventricular repolarization and sudden cardiac death (SCD; Ip *et al*, 2020). The cardiotoxic effects of e‐cigarettes on channels could increase the likelihood of sudden cardiac death in individuals with mutations in ion channels. iPSC disease modeling in conjunction with novel gene‐editing technologies (Nishiga *et al*, 2022) are being deployed to uncover the possible causal link and identify individuals who may be at a greater risk of SCD after e‐cigarette exposure, as well as possibly identify novel antiarrhythmic agents that might prevent these adverse outcomes.

EVALI underscores the importance of testing e‐cigarette devices before marketing the products to the public. Clinical investigations using bronchoalveolar lavages indicated that nicotine, Δ^9^‐tetrahydrocannabinol (Δ^9^‐THC), the psychedelic component of marijuana, and vitamin E acetate, a component used to emulsify Δ^9^‐THC, were linked to EVALI. Studies in mouse models have found conflicting results, with one study suggesting vitamin E acetate is the causative agent. A more recent study indicates that the vehicle vegetable glycerol and propylene glycol might be sufficient to cause injury (Blount *et al*, 2019). iPSC‐derived lung tissue may be used to provide a more exhaustive study of the components of e‐cigarettes that cause EVALI.

## Marijuana

Associated with EVALI, marijuana is emerging as a new potential and growing threat to cardiopulmonary health. The most popular illicit drug in the world is becoming legalized for medicinal and recreational uses, with rapidly growing rates of marijuana use being reported with legalization (Cerda *et al*, 2020). The long‐term effects of marijuana remain unclear because of years of restrictions on research, but it is implicated in cardiomyopathy, arrhythmias, and vascular disease from case reports and case series (Pacher *et al*, 2018). A recent large epidemiological study suggests that the odds ratio of marijuana is greater than traditional cigarettes and can predispose younger patients to premature cardiovascular disease (DeFilippis *et al*, 2018; Wei *et al*, 2022).

With legalization, the use of marijuana will increase and expose larger segments of the population to its potentially adverse effects (Pacher & Gao, 2008). Marijuana affects the body via the cannabinoid receptor 1 (CB1) and cannabinoid receptor 2 (CB2; Pacher *et al*, 2018). CB1 activation is associated with endothelial dysfunction and atherosclerosis (El‐Remessy *et al*, 2011; Rajesh *et al*, 2012), whereas CB2 activation is related to vascular quiescence (Pacher & Mackie, 2012; Pacher *et al*, 2018). Marijuana is composed of over a hundred different cannabinoids. The psychedelic component of marijuana, Δ^9^‐THC, is an agonist of the CB1 receptor and causes inflammation and oxidative stress (El‐Remessy *et al*, 2011; Pacher *et al*, 2018). iPSC disease modeling and CRISPR‐Cas9 gene editing were used to elucidate the mechanisms Δ^9^‐THC‐mediated vascular dysfunction (Wei *et al*, 2022).

Δ^9^‐THC promotes inflammation and oxidative stress via the CB1 receptor, and the mechanisms have been previously described for MAP kinase activation and NF‐kb pathways. Briefly, CB1 mediates increased oxidative stress and inflammation implicated in diabetic retinopathy, cardiomyopathy, and endothelial dysfunction (Mukhopadhyay *et al*, 2010; Rajesh *et al*, 2010a; El‐Remessy *et al*, 2011; Rajesh *et al*, 2012). CB1 activation occurs via the MAP kinase pathway, which causes oxidative stress, inflammation, and cell death in human coronary artery endothelial cells (Liu *et al*, 2000; Pertwee *et al*, 2010; Rajesh *et al*, 2010b). iPSC disease modeling could elucidate the mechanisms accounting for why some individuals are more likely to develop cardiovascular disease or arrhythmias after using cannabinoids. While these studies in primary cells are important for understanding mechanisms of cardiovascular disease, iPSC disease modeling with vascular cells can also be used to reveal novel mechanisms that might serve as biomarkers and lead to novel therapies for the disease. Moreover, patient‐specific polymorphisms may help predict which individuals are more susceptible to adverse effects of marijuana or smoking or vaping. iPSC disease modeling could uncover polymorphisms that make an individual more likely to develop cardiovascular disease with cannabis exposure.

Some recent studies in iPSC‐derived neurons suggest that THC exposure perturbs gene expression profiles and is linked to neuropsychiatric disorders such as schizophrenia (Noh *et al*, 2017; Guennewig *et al*, 2018). iPSC‐embryoid body toxicity profiling is likely to reveal more global adverse effects of cannabinoids with the use of transcriptional profiling. Because it is considered an herbal remedy for nausea and vomiting, cannabis is often used by pregnant women, and epidemiological studies on prenatal exposure to cannabis indicated that it is associated with psychiatric disorders of offspring (Roncero *et al*, 2020). Recent investigations in iPSC‐derived neurons have described neurotoxicity caused by physiologic doses of THC exposure (10 μM) and perturbations in the expression of voltage‐gated calcium channels (Miranda *et al*, 2020). Indeed, marijuana is associated with cardiac arrhythmias, and iPSC disease modeling can be used to elucidate the molecular mechanisms of how cannabinoids might cause arrhythmias. The long‐term effects of marijuana on the cardiovascular system are not completely understood. However, a recent study showed using UK Biobank data that marijuana was a risk factor for cardiovascular disease (Wei *et al*, 2022). Here Wei *et al* (2022) used iPSC disease modeling and small animal models to investigate the mechanisms of Δ^9^‐THC‐mediated vascular dysfunction via the CB1 receptor. The prevalence of marijuana use is expected to increase with its growing legalization, and is likely to cause additional unanticipated, adverse health effects such as EVALI. Therefore, there is an urgent need to study the impact of marijuana in a pre‐clinical model that is more translatable and relevant to the human body than existing cell culture and animal studies.

## Opioids

The current opioid epidemic in the United States has developed in three phases. In the 1990s, most of the opioid‐related deaths were caused by increased prescriptions of natural and semi‐synthetic opioids. A second wave began in 2010 with high usage of heroin (Rudd *et al*, 2016). The latest wave of opioid‐related deaths is associated with the significant increase in use of illicitly manufactured fentanyl. Overdose‐related deaths are mainly caused by bradycardia and respiratory depression. While recently oxycodone and hydrocodone have been the most commonly used opioids involved in overdose deaths (Mattson *et al*, 2021), more recently fentanyl and its derivatives are involved in more cardiovascular complications and malignant arrhythmias. Methadone has been well described to inhibit human cardiac ether‐a‐go‐go‐related gene (hERG)‐associated K+ current, the rapid component of the delayed rectifier current (IKr), which determines the duration of the resting QT interval (Katchman *et al*, 2002). Inhibition of this current results in a prolonged cardiac repolarization phase of the action potential, extending the QT interval and increasing the vulnerability to arrhythmias (Roden, 2004). Nevertheless, the ambulatory data on methadone‐induced cardiac arrests are limited, and perioperative data on QTc prolongation in patients undergoing non‐cardiac surgery suggest that even after administration of more than nine QTc‐prolonging drugs, opioids do not significantly contribute to clinically relevant QTc prolongation (Roden, 2004; Obal *et al*, 2014).

A more subtle effect may be occurring on the cardiovascular system. Epidemiological data suggest that chronic opioid administration is associated with a higher incidence of myocardial infarction and stroke (Chen & Ashburn, 2015). The American Heart Association (AHA) recently advised on the effect of chronic exposure to synthetic opioid analogs on cardiovascular (CV) function underscoring their potential detrimental impact on CV function and health (Chow *et al*, 2021; Dezfulian *et al*, 2021). Surprisingly, little is known about how chronic opioids affect endothelial and cardiomyocyte function. Distracted by the apparent short‐term effects of opioids (i.e., respiratory depression and bowel obstruction), long‐term effects have been lurking in the shadows and unrecognized. Recent insights into opioid receptor function and binding of different ligands have provided a broader understanding on the complex regulation of opioid signaling within the cardiovascular system (Gladden *et al*, 2016; O'Donnell & Jackson, 2017).

## Microplastics

The widespread use of plastics and microplastics is a potentially novel hazard (Matthews *et al*, 2021). A recent study revealed that the abundance of microplastics may be vastly underestimated, with over 5 trillion pieces of plastic in the world's oceans that have a combined mass of 250,000 tons (Eriksen *et al*, 2014). The United Nations Environment Assembly (UNEP) has estimated that 4.8–12.7 million tons of plastic are introduced into oceans annually (Haward, 2018). The long‐term consequences of such ubiquitous pollution with plastics on the body are unknown but increasingly found in all elements of the aquatic food chain (Lehel & Murphy, 2021).

By studying the effects of microplastics *in vitro*, iPSCs might provide a window into the effects of plastics. Microplastics are consumed by plankton and are found in all aquatic species (Lehel & Murphy, 2021). The largest source of protein for humans is fish, and the microplastics do not simply transit through the gastrointestinal system but instead accumulate in the circulatory and adipose tissues (Lehel & Murphy, 2021). iPSC disease modeling can be used to understand how microplastics affect the gastrointestinal system, central nervous system, and the cardiovascular system. For example, epigenetic changes and the introduction on mutations in the genetic code may herald the initiation and progression of carcinogenesis. Initial studies with iPSCs have only revealed growth inhibition (Jeong *et al*, 2018). More recent studies have employed organoid models to understand how microplastics affect the body (Miloradovic *et al*, 2021; Winkler *et al*, 2022). Indeed, plastics are likely to modify gene expression and cause disease. More worrisome, plastics may alter epigenetic expression and be transmitted to future generations.

## Summary and conclusion

iPSC disease modeling is emerging as a powerful paradigm for understanding the patient‐specific disease mechanisms. The interface with gene‐editing tools and iPSCs allows investigators to elucidate the underlying molecular mechanisms that cause disease. iPSCs, gene‐editing tools, and transcriptomics have the potential to revolutionize toxicology and rapidly advance our understanding of the adverse effects of environmental toxins. Because they contain the genetic code unique to each person, the use of iPSCs promises the discovery of new gene–environment interactions that can decipher why some individuals are more susceptible to environmental factors that exacerbate diseases such as cardiovascular disease.

## Author contributions

**Joseph C Wu:** Conceptualization; resources; supervision; writing – original draft; writing – review and editing. **Mark Chandy:** Conceptualization; visualization; writing – original draft; writing – review and editing. **Detlef Obal:** Conceptualization; writing – original draft; writing – review and editing.

In addition to the CRediT author contributions listed above, the contributions in detail are:

MC: manuscript writing and preparing figures; DO: manuscript writing; JCW: manuscript writing and final approval of the manuscript.

## Disclosure and competing interests statement

J.C.W. is a co‐founder and SAB of Greenstone Biosciences, and M.C. is a consultant for Greenstone Biosciences, but this manuscript was written independently.

**Pending issues**

iPSCs are a powerful model system to investigate the adverse effects of these compounds on the body and avoid animal testing. However, 2D iPSC‐derived tissues are immature and may not fully recapitulate the cellular physiology of somatic cells. With the development of 3D models such as organoids and EHT, and advances in single‐cell RNA sequencing, iPSC disease modeling has improved significantly. 3D models provide a better environment that promotes cellular maturation and development. More importantly, single‐cell RNA sequencing can provide spatial transcriptomics of complex cell–cell interactions. With ongoing advancements in biomaterials, biomedical engineering, and next‐generation sequencing, iPSC disease modeling is continuing to evolve into a high‐throughput screening platform for environmental exposures.

**For more information**

For additional information on iPSC Biobanking, cell culture protocols, publications, and contact information to request iPSC lines, please visit the Stanford Cardiovascular Institute (SCVI) Biobank website: https://med.stanford.edu/scvibiobank.html.

## References

[emmm202013260-bib-0001] Ambrose JA , Barua RS (2004) The pathophysiology of cigarette smoking and cardiovascular disease: an update. J Am Coll Cardiol 43: 1731–1737 15145091 10.1016/j.jacc.2003.12.047

[emmm202013260-bib-0002] Asimaki A , Kapoor S , Plovie E , Karin Arndt A , Adams E , Liu Z , James CA , Judge DP , Calkins H , Churko J *et al* (2014) Identification of a new modulator of the intercalated disc in a zebrafish model of arrhythmogenic cardiomyopathy. Sci Transl Med 6: 240ra274 10.1126/scitranslmed.3008008 PMC4471875 24920660

[emmm202013260-bib-0003] Benowitz NL , Fraiman JB (2017) Cardiovascular effects of electronic cigarettes. Nat Rev Cardiol 14: 447–456 28332500 10.1038/nrcardio.2017.36 PMC5519136

[emmm202013260-bib-0004] Beqqali A , van Eldik W , Mummery C , Passier R (2009) Human stem cells as a model for cardiac differentiation and disease. Cell Mol Life Sci 66: 800–813 19151924 10.1007/s00018-009-8476-0 PMC11131531

[emmm202013260-bib-0005] Bhatnagar A (2017) Environmental determinants of cardiovascular disease. Circ Res 121: 162–180 28684622 10.1161/CIRCRESAHA.117.306458 PMC5777598

[emmm202013260-bib-0006] Blount BC , Karwowski MP , Shields PG , Morel‐Espinosa M , Valentin‐Blasini L , Gardner M , Braselton M , Brosius CR , Caron KT , Chambers D *et al* (2019) Vitamin E acetate in bronchoalveolar‐lavage fluid associated with EVALI. N Engl J Med 382: 697–705 31860793 10.1056/NEJMoa1916433 PMC7032996

[emmm202013260-bib-0007] Brook RD , Franklin B , Cascio W , Hong Y , Howard G , Lipsett M , Luepker R , Mittleman M , Samet J , Smith SC Jr *et al* (2004) Air pollution and cardiovascular disease: A statement for healthcare professionals from the expert panel on population and prevention science of the American Heart Association. Circulation 109: 2655–2671 15173049 10.1161/01.CIR.0000128587.30041.C8

[emmm202013260-bib-0008] Cai B , Xia T , Qian Y , Lu H , Cai R , Wang C (2020) Association between fine particulate matter and fatal hemorrhagic stroke incidence: a time stratified case‐crossover study in Shanghai, China. J Occup Environ Med 62: 916–921 32769785 10.1097/JOM.0000000000001973

[emmm202013260-bib-0009] Carvajal‐Vergara X , Sevilla A , D'Souza SL , Ang YS , Schaniel C , Lee DF , Yang L , Kaplan AD , Adler ED , Rozov R *et al* (2010) Patient‐specific induced pluripotent stem‐cell‐derived models of LEOPARD syndrome. Nature 465: 808–812 20535210 10.1038/nature09005 PMC2885001

[emmm202013260-bib-0010] Cerda M , Mauro C , Hamilton A , Levy NS , Santaella‐Tenorio J , Hasin D , Wall MM , Keyes KM , Martins SS (2020) Association between recreational marijuana legalization in the United States and changes in marijuana use and cannabis use disorder from 2008 to 2016. JAMA Psychiat 77: 165–171 10.1001/jamapsychiatry.2019.3254 PMC6865220 31722000

[emmm202013260-bib-0011] Chen A , Ashburn MA (2015) Cardiac effects of opioid therapy. Pain Med 16(Suppl 1): S27‐31 26461073 10.1111/pme.12915

[emmm202013260-bib-0012] Chow SL , Sasson C , Benjamin IJ , Califf RM , Compton WM , Oliva EM , Robson C , Sanchez EJ (2021) Opioid use and its relationship to cardiovascular disease and brain health: a presidential advisory from the American Heart Association. Circulation 144: e218–e232 34407637 10.1161/CIR.0000000000001007

[emmm202013260-bib-0013] Collaborators GBDT (2017) Smoking prevalence and attributable disease burden in 195 countries and territories, 1990‐2015: a systematic analysis from the global burden of disease study 2015. Lancet 389: 1885–1906 28390697 10.1016/S0140-6736(17)30819-X PMC5439023

[emmm202013260-bib-0014] Czyz J , Guan K , Zeng Q , Nikolova T , Meister A , Schonborn F , Schuderer J , Kuster N , Wobus AM (2004a) High frequency electromagnetic fields (GSM signals) affect gene expression levels in tumor suppressor p53‐deficient embryonic stem cells. Bioelectromagnetics 25: 296–307 15114639 10.1002/bem.10199

[emmm202013260-bib-0015] Czyz J , Nikolova T , Schuderer J , Kuster N , Wobus AM (2004b) Non‐thermal effects of power‐line magnetic fields (50 Hz) on gene expression levels of pluripotent embryonic stem cells‐the role of tumour suppressor p53. Mutat Res 557: 63–74 14706519 10.1016/j.mrgentox.2003.09.011

[emmm202013260-bib-0016] DeFilippis EM , Singh A , Divakaran S , Gupta A , Collins BL , Biery D , Qamar A , Fatima A , Ramsis M , Pipilas D *et al* (2018) Cocaine and marijuana use among young adults with myocardial infarction. J Am Coll Cardiol 71: 2540–2551 29535062 10.1016/j.jacc.2018.02.047 PMC6495189

[emmm202013260-bib-0017] Dezfulian C , Orkin AM , Maron BA , Elmer J , Girotra S , Gladwin MT , Merchant RM , Panchal AR , Perman SM , Starks MA *et al* (2021) Opioid-associated out-of-hospital cardiac arrest: distinctive clinical features and implications for health care and public responses: a scientific statement from the American Heart Association. Circulation 143: e836–e870 33682423 10.1161/CIR.0000000000000958

[emmm202013260-bib-0018] Drummond MB , Upson D (2014) Electronic cigarettes. Potential harms and benefits. Ann Am Thorac Soc 11: 236–242 24575993 10.1513/AnnalsATS.201311-391FR PMC5469426

[emmm202013260-bib-0019] El‐Remessy AB , Rajesh M , Mukhopadhyay P , Horvath B , Patel V , Al‐Gayyar MM , Pillai BA , Pacher P (2011) Cannabinoid 1 receptor activation contributes to vascular inflammation and cell death in a mouse model of diabetic retinopathy and a human retinal cell line. Diabetologia 54: 1567–1578 21373835 10.1007/s00125-011-2061-4 PMC3375271

[emmm202013260-bib-0020] Eriksen M , Lebreton LC , Carson HS , Thiel M , Moore CJ , Borerro JC , Galgani F , Ryan PG , Reisser J (2014) Plastic pollution in the World's oceans: more than 5 trillion plastic pieces weighing over 250,000 tons afloat at sea. PLoS One 9: e111913 25494041 10.1371/journal.pone.0111913 PMC4262196

[emmm202013260-bib-0021] Feng C , Li J , Sun W , Zhang Y , Wang Q (2016) Impact of ambient fine particulate matter (PM_2.5_) exposure on the risk of influenza‐like‐illness: a time‐series analysis in Beijing, China. Environ Health 15: 17 26864833 10.1186/s12940-016-0115-2 PMC4750357

[emmm202013260-bib-0022] Gladden RM , Martinez P , Seth P (2016) Fentanyl law enforcement submissions and increases in synthetic opioid‐involved overdose deaths ‐ 27 states, 2013‐2014. MMWR Morb Mortal Wkly Rep 65: 837–843 27560775 10.15585/mmwr.mm6533a2

[emmm202013260-bib-0023] Grskovic M , Javaherian A , Strulovici B , Daley GQ (2011) Induced pluripotent stem cells: opportunities for disease modelling and drug discovery. Nat Rev Drug Discov 10: 915–929 22076509 10.1038/nrd3577

[emmm202013260-bib-0024] Guennewig B , Bitar M , Obiorah I , Hanks J , O'Brien EA , Kaczorowski DC , Hurd YL , Roussos P , Brennand KJ , Barry G (2018) THC exposure of human iPSC neurons impacts genes associated with neuropsychiatric disorders. Transl Psychiatry 8: 89 29691375 10.1038/s41398-018-0137-3 PMC5915454

[emmm202013260-bib-0025] Guo H , Tian L , Zhang JZ , Kitani T , Paik DT , Lee WH , Wu JC (2019) Single‐cell RNA sequencing of human embryonic stem cell differentiation delineates adverse effects of nicotine on embryonic development. Stem Cell Reports 12: 772–786 30827876 10.1016/j.stemcr.2019.01.022 PMC6449785

[emmm202013260-bib-0026] van der Harst P , Verweij N (2018) Identification of 64 novel genetic loci provides an expanded view on the genetic architecture of coronary artery disease. Circ Res 122: 433–443 29212778 10.1161/CIRCRESAHA.117.312086 PMC5805277

[emmm202013260-bib-0027] Haward M (2018) Plastic pollution of the world's seas and oceans as a contemporary challenge in ocean governance. Nat Commun 9: 667 29445166 10.1038/s41467-018-03104-3 PMC5812987

[emmm202013260-bib-0028] Hindy G , Wiberg F , Almgren P , Melander O , Orho‐Melander M (2018) Polygenic risk score for coronary heart disease modifies the elevated risk by cigarette smoking for disease incidence. Circ Genom Precis Med 11: e001856 29874179 10.1161/CIRCGEN.117.001856 PMC6319562

[emmm202013260-bib-0029] Hoek G , Krishnan RM , Beelen R , Peters A , Ostro B , Brunekreef B , Kaufman JD (2013) Long‐term air pollution exposure and cardio‐ respiratory mortality: a review. Environ Health 12: 43 23714370 10.1186/1476-069X-12-43 PMC3679821

[emmm202013260-bib-0030] Hsu PD , Lander ES , Zhang F (2014) Development and applications of CRISPR‐Cas9 for genome engineering. Cell 157: 1262–1278 24906146 10.1016/j.cell.2014.05.010 PMC4343198

[emmm202013260-bib-0031] Ip M , Diamantakos E , Haptonstall K , Choroomi Y , Moheimani RS , Nguyen KH , Tran E , Gornbein J , Middlekauff HR (2020) Tobacco and electronic cigarettes adversely impact ECG indexes of ventricular repolarization: Implication for sudden death risk. Am J Physiol Heart Circ Physiol 318: H1176–H1184 32196360 10.1152/ajpheart.00738.2019 PMC7346537

[emmm202013260-bib-0032] Jalali Z , Bahrampour S , Khalili P , Khademalhosseini M , Esmaeili Nadimi A (2021) Cohort‐based analysis of paternal opioid use in relation to offspring's BMI and plasma lipid profile. Sci Rep 11: 9462 33947903 10.1038/s41598-021-88781-9 PMC8096835

[emmm202013260-bib-0033] Jeong CB , Kang HM , Lee YH , Kim MS , Lee JS , Seo JS , Wang M , Lee JS (2018) Nanoplastic ingestion enhances toxicity of persistent organic pollutants (POPs) in the monogonont rotifer Brachionus koreanus via multixenobiotic resistance (MXR) disruption. Environ Sci Technol 52: 11411–11418 30192528 10.1021/acs.est.8b03211

[emmm202013260-bib-0034] Johnson HM , Gossett LK , Piper ME , Aeschlimann SE , Korcarz CE , Baker TB , Fiore MC , Stein JH (2010) Effects of smoking and smoking cessation on endothelial function: 1‐year outcomes from a randomized clinical trial. J Am Coll Cardiol 55: 1988–1995 20236788 10.1016/j.jacc.2010.03.002 PMC2947952

[emmm202013260-bib-0035] Jones CG , Rappold AG , Vargo J , Cascio WE , Kharrazi M , McNally B , Hoshiko S , with the CARES Surveillance Group (2020) Out‐of‐hospital cardiac arrests and wildfire‐related particulate matter during 2015‐2017 California wildfires. J Am Heart Assoc 9: e014125 32290746 10.1161/JAHA.119.014125 PMC7428528

[emmm202013260-bib-0036] Karakikes I , Termglinchan V , Cepeda DA , Lee J , Diecke S , Hendel A , Itzhaki I , Ameen M , Shrestha R , Wu H *et al* (2017) A comprehensive TALEN‐based knockout library for generating human‐induced pluripotent stem cell‐based models for cardiovascular diseases. Circ Res 120: 1561–1571 28246128 10.1161/CIRCRESAHA.116.309948 PMC5429194

[emmm202013260-bib-0037] Katchman AN , McGroary KA , Kilborn MJ , Kornick CA , Manfredi PL , Woosley RL , Ebert SN (2002) Influence of opioid agonists on cardiac human ether‐a‐go‐go‐related gene K(+) currents. J Pharmacol Exp Ther 303: 688–694 12388652 10.1124/jpet.102.038240

[emmm202013260-bib-0038] Kawamura M , Miyagawa S , Miki K , Saito A , Fukushima S , Higuchi T , Kawamura T , Kuratani T , Daimon T , Shimizu T *et al* (2012) Feasibility, safety, and therapeutic efficacy of human induced pluripotent stem cell‐derived cardiomyocyte sheets in a porcine ischemic cardiomyopathy model. Circulation 126: S29–S37 22965990 10.1161/CIRCULATIONAHA.111.084343

[emmm202013260-bib-0039] Kim C , Wong J , Wen J , Wang S , Wang C , Spiering S , Kan NG , Forcales S , Puri PL , Leone TC *et al* (2013) Studying arrhythmogenic right ventricular dysplasia with patient‐specific iPSCs. Nature 494: 105–110 23354045 10.1038/nature11799 PMC3753229

[emmm202013260-bib-0040] Kim H , Kim WH , Kim YY , Park HY (2020a) Air pollution and central nervous system disease: A review of the impact of fine particulate matter on neurological disorders. Front Public Health 8: 575330 33392129 10.3389/fpubh.2020.575330 PMC7772244

[emmm202013260-bib-0041] Kim IS , Yang PS , Lee J , Yu HT , Kim TH , Uhm JS , Kim JY , Pak HN , Lee MH , Joung B (2020b) Long‐term fine particulate matter exposure and cardiovascular mortality in the general population: a nationwide cohort study. J Cardiol 75: 549–558 31839460 10.1016/j.jjcc.2019.11.004

[emmm202013260-bib-0042] Kim OJ , Lee SH , Kang SH , Kim SY (2020c) Incident cardiovascular disease and particulate matter air pollution in South Korea using a population‐based and nationwide cohort of 0.2 million adults. Environ Health 19: 113 33167999 10.1186/s12940-020-00671-1 PMC7653702

[emmm202013260-bib-0043] Kim H , Kamm RD , Vunjak‐Novakovic G , Wu JC (2022) Progress in multicellular human cardiac organoids for clinical applications. Cell Stem Cell 29: 503–514 35395186 10.1016/j.stem.2022.03.012 PMC9352318

[emmm202013260-bib-0044] Kodo K , Ong SG , Jahanbani F , Termglinchan V , Hirono K , InanlooRahatloo K , Ebert AD , Shukla P , Abilez OJ , Churko JM *et al* (2016) iPSC‐derived cardiomyocytes reveal abnormal TGF‐beta signalling in left ventricular non‐compaction cardiomyopathy. Nat Cell Biol 18: 1031–1042 27642787 10.1038/ncb3411 PMC5042877

[emmm202013260-bib-0045] Lan F , Lee AS , Liang P , Sanchez‐Freire V , Nguyen PK , Wang L , Han L , Yen M , Wang Y , Sun N *et al* (2013) Abnormal calcium handling properties underlie familial hypertrophic cardiomyopathy pathology in patient‐specific induced pluripotent stem cells. Cell Stem Cell 12: 101–113 23290139 10.1016/j.stem.2012.10.010 PMC3638033

[emmm202013260-bib-0046] Larsson SC , Mason AM , Back M , Klarin D , Damrauer SM , Million Veteran P , Michaelsson K , Burgess S (2020) Genetic predisposition to smoking in relation to 14 cardiovascular diseases. Eur Heart J 41: 3304–3310 32300774 10.1093/eurheartj/ehaa193 PMC7544540

[emmm202013260-bib-0047] Lau E , Paik DT , Wu JC (2019) Systems‐wide approaches in induced pluripotent stem cell models. Annu Rev Pathol 14: 395–419 30379619 10.1146/annurev-pathmechdis-012418-013046 PMC6450651

[emmm202013260-bib-0048] Lee WH , Ong S‐G , Zhou Y , Tian L , Bae HR , Baker N , Whitlatch A , Mohammadi L , Guo H , Nadeau KC *et al* (2019) Modeling cardiovascular risks of E‐cigarettes with human‐induced pluripotent stem cell–derived endothelial cells. J Am Coll Cardiol 73: 2722–2737 31146818 10.1016/j.jacc.2019.03.476 PMC6637948

[emmm202013260-bib-0049] Lehel J , Murphy S (2021) Microplastics in the food chain: Food safety and environmental aspects. Rev Environ Contam Toxicol 259: 1–49 34611754 10.1007/398_2021_77

[emmm202013260-bib-0050] Levin MG , Klarin D , Assimes TL , Freiberg MS , Ingelsson E , Lynch J , Natarajan P , O'Donnell C , Rader DJ , Tsao PS *et al* (2021) Genetics of smoking and risk of atherosclerotic cardiovascular diseases: a mendelian randomization study. JAMA Netw Open 4: e2034461 33464320 10.1001/jamanetworkopen.2020.34461 PMC7816104

[emmm202013260-bib-0051] Liu J , Gao B , Mirshahi F , Sanyal AJ , Khanolkar AD , Makriyannis A , Kunos G (2000) Functional CB1 cannabinoid receptors in human vascular endothelial cells. Biochem J 346: 835–840 10698714 PMC1220920

[emmm202013260-bib-0052] Lu TY , Lin B , Kim J , Sullivan M , Tobita K , Salama G , Yang L (2013) Repopulation of decellularized mouse heart with human induced pluripotent stem cell‐derived cardiovascular progenitor cells. Nat Commun 4: 2307 23942048 10.1038/ncomms3307

[emmm202013260-bib-0053] Luo YS , Chen Z , Blanchette AD , Zhou YH , Wright FA , Baker ES , Chiu WA , Rusyn I (2021) Relationships between constituents of energy drinks and beating parameters in human induced pluripotent stem cell (iPSC)‐derived cardiomyocytes. Food Chem Toxicol 149: 111979 33450301 10.1016/j.fct.2021.111979 PMC8286543

[emmm202013260-bib-0054] Ma N , Zhang JZ , Itzhaki I , Zhang SL , Chen H , Haddad F , Kitani T , Wilson KD , Tian L , Shrestha R *et al* (2018) Determining the pathogenicity of a genomic variant of uncertain significance using CRISPR/Cas9 and human‐induced pluripotent stem cells. Circulation 138: 2666–2681 29914921 10.1161/CIRCULATIONAHA.117.032273 PMC6298866

[emmm202013260-bib-0055] Matsa E , Denning C (2012) In vitro uses of human pluripotent stem cell‐derived cardiomyocytes. J Cardiovasc Transl Res 5: 581–592 22639342 10.1007/s12265-012-9376-5

[emmm202013260-bib-0056] Matthews S , Mai L , Jeong CB , Lee JS , Zeng EY , Xu EG (2021) Key mechanisms of micro‐ and nanoplastic (MNP) toxicity across taxonomic groups. Comp Biochem Physiol C Toxicol Pharmacol 247: 109056 33894368 10.1016/j.cbpc.2021.109056

[emmm202013260-bib-0057] Mattson CL , Tanz LJ , Quinn K , Kariisa M , Patel P , Davis NL (2021) Trends and geographic patterns in drug and synthetic opioid overdose deaths ‐ United States, 2013‐2019. MMWR Morb Mortal Wkly Rep 70: 202–207 33571180 10.15585/mmwr.mm7006a4 PMC7877587

[emmm202013260-bib-0058] Miloradovic D , Pavlovic D , Jankovic MG , Nikolic S , Papic M , Milivojevic N , Stojkovic M , Ljujic B (2021) Human embryos, induced pluripotent stem cells, and organoids: models to assess the effects of environmental plastic pollution. Front Cell Dev Biol 9: 709183 34540831 10.3389/fcell.2021.709183 PMC8446652

[emmm202013260-bib-0059] Miranda CC , Barata T , Vaz SH , Ferreira C , Quintas A , Bekman EP (2020) hiPSC‐based model of prenatal exposure to cannabinoids: effect on neuronal differentiation. Front Mol Neurosci 13: 119 32733202 10.3389/fnmol.2020.00119 PMC7357827

[emmm202013260-bib-0060] Mukhopadhyay P , Rajesh M , Batkai S , Patel V , Kashiwaya Y , Liaudet L , Evgenov OV , Mackie K , Hasko G , Pacher P (2010) CB1 cannabinoid receptors promote oxidative stress and cell death in murine models of doxorubicin‐induced cardiomyopathy and in human cardiomyocytes. Cardiovasc Res 85: 773–784 19942623 10.1093/cvr/cvp369 PMC2819835

[emmm202013260-bib-0061] Munzel T , Hahad O , Kuntic M , Keaney JF , Deanfield JE , Daiber A (2020) Effects of tobacco cigarettes, e‐cigarettes, and waterpipe smoking on endothelial function and clinical outcomes. Eur Heart J 41: 4057–4070 32585699 10.1093/eurheartj/ehaa460 PMC7454514

[emmm202013260-bib-0062] Nelson TJ , Martinez‐Fernandez A , Yamada S , Perez‐Terzic C , Ikeda Y , Terzic A (2009) Repair of acute myocardial infarction by human stemness factors induced pluripotent stem cells. Circulation 120: 408–416 19620500 10.1161/CIRCULATIONAHA.109.865154 PMC2768575

[emmm202013260-bib-0063] Neofytou E , O'Brien CG , Couture LA , Wu JC (2015) Hurdles to clinical translation of human induced pluripotent stem cells. J Clin Invest 125: 2551–2557 26132109 10.1172/JCI80575 PMC4563685

[emmm202013260-bib-0064] Nikolova T , Czyz J , Rolletschek A , Blyszczuk P , Fuchs J , Jovtchev G , Schuderer J , Kuster N , Wobus AM (2005) Electromagnetic fields affect transcript levels of apoptosis‐related genes in embryonic stem cell‐derived neural progenitor cells. FASEB J 19: 1686–1688 16116041 10.1096/fj.04-3549fje

[emmm202013260-bib-0065] Nishiga M , Liu C , Qi LS , Wu JC (2022) The use of new CRISPR tools in cardiovascular research and medicine. Nat Rev Cardiol 19: 505–521 35145236 10.1038/s41569-021-00669-3 PMC10283450

[emmm202013260-bib-0066] Noh H , Shao Z , Coyle JT , Chung S (2017) Modeling schizophrenia pathogenesis using patient‐derived induced pluripotent stem cells (iPSCs). Biochim Biophys Acta Mol Basis Dis 1863: 2382–2387 28668333 10.1016/j.bbadis.2017.06.019 PMC5737829

[emmm202013260-bib-0067] Nystoriak MA , Kilfoil PJ , Lorkiewicz PK , Ramesh B , Kuehl PJ , McDonald J , Bhatnagar A , Conklin DJ (2019) Comparative effects of parent and heated cinnamaldehyde on the function of human iPSC‐derived cardiac myocytes. Toxicol In Vitro 61: 104648 31518667 10.1016/j.tiv.2019.104648 PMC7278494

[emmm202013260-bib-0068] Obal D , Wu JC (2020) Induced pluripotent stem cells as a platform to understand patient‐specific responses to opioids and anaesthetics. Br J Pharmacol 177: 4581–4594 32767563 10.1111/bph.15228 PMC7520445

[emmm202013260-bib-0069] Obal D , Yang D , Sessler DI (2014) Perioperative doses of ondansetron or dolasetron do not lengthen the QT interval. Mayo Clin Proc 89: 69–80 24388024 10.1016/j.mayocp.2013.10.008

[emmm202013260-bib-0070] O'Donnell FT , Jackson DL (2017) Opioid use disorder and pregnancy. Mo Med 114: 181–186 30228577 PMC6140233

[emmm202013260-bib-0071] Pacher P , Gao B (2008) Endocannabinoids and liver disease. III. Endocannabinoid effects on immune cells: Implications for inflammatory liver diseases. Am J Physiol Gastrointest Liver Physiol 294: G850–G854 18239059 10.1152/ajpgi.00523.2007 PMC2376822

[emmm202013260-bib-0072] Pacher P , Mackie K (2012) Interplay of cannabinoid 2 (CB2) receptors with nitric oxide synthases, oxidative and nitrative stress, and cell death during remote neurodegeneration. J Mol Med 90: 347–351 22371074 10.1007/s00109-012-0884-1

[emmm202013260-bib-0073] Pacher P , Steffens S , Hasko G , Schindler TH , Kunos G (2018) Cardiovascular effects of marijuana and synthetic cannabinoids: the good, the bad, and the ugly. Nat Rev Cardiol 15: 151–166 28905873 10.1038/nrcardio.2017.130

[emmm202013260-bib-0074] Paik DT , Chandy M , Wu JC (2020) Patient and disease‐specific induced pluripotent stem cells for discovery of personalized cardiovascular drugs and therapeutics. Pharmacol Rev 72: 320–342 31871214 10.1124/pr.116.013003 PMC6934989

[emmm202013260-bib-0075] Pertwee RG , Howlett AC , Abood ME , Alexander SP , Di Marzo V , Elphick MR , Greasley PJ , Hansen HS , Kunos G , Mackie K *et al* (2010) International union of basic and clinical pharmacology. LXXIX. Cannabinoid receptors and their ligands: Beyond CB(1) and CB(2). Pharmacol Rev 62: 588–631 21079038 10.1124/pr.110.003004 PMC2993256

[emmm202013260-bib-0076] Polosa R , Caponnetto P (2016) The health effects of electronic cigarettes. N Engl J Med 375: 2608–2609 10.1056/NEJMc1613869 28032962

[emmm202013260-bib-0077] Rajagopalan S , Brauer M , Bhatnagar A , Bhatt DL , Brook JR , Huang W , Munzel T , Newby D , Siegel J , Brook RD *et al* (2020) Personal‐level protective actions against particulate matter air pollution exposure: a scientific statement from the American Heart Association. Circulation 142: e411–e431 33150789 10.1161/CIR.0000000000000931

[emmm202013260-bib-0078] Rajesh M , Mukhopadhyay P , Bátkai S , Patel V , Saito K , Matsumoto S , Kashiwaya Y , Horváth B , Mukhopadhyay B , Becker L *et al* (2010a) Cannabidiol attenuates cardiac dysfunction, oxidative stress, fibrosis, and inflammatory and cell death signaling pathways in diabetic cardiomyopathy. J Am Coll Cardiol 56: 2115–2125 21144973 10.1016/j.jacc.2010.07.033 PMC3026637

[emmm202013260-bib-0079] Rajesh M , Mukhopadhyay P , Hasko G , Liaudet L , Mackie K , Pacher P (2010b) Cannabinoid‐1 receptor activation induces reactive oxygen species‐dependent and ‐independent mitogen‐activated protein kinase activation and cell death in human coronary artery endothelial cells. Br J Pharmacol 160: 688–700 20590572 10.1111/j.1476-5381.2010.00712.x PMC2931568

[emmm202013260-bib-0080] Rajesh M , Batkai S , Kechrid M , Mukhopadhyay P , Lee WS , Horvath B , Holovac E , Cinar R , Liaudet L , Mackie K *et al* (2012) Cannabinoid 1 receptor promotes cardiac dysfunction, oxidative stress, inflammation, and fibrosis in diabetic cardiomyopathy. Diabetes 61: 716–727 22315315 10.2337/db11-0477 PMC3282820

[emmm202013260-bib-0081] Roden DM (2004) Antiarrhythmic drugs: Past, present, and future. Heart Rhythm 1: 57C–66C 10.1016/j.hrthm.2004.10.019 23570108

[emmm202013260-bib-0082] Rohani F , Rezayat AA , Zarifian A , Nour MG , Vakilian F , Sahebkar A , Dadgarmoghaddam M (2021) Opioid dependency and myocardial infarction: a systematic review and meta‐analysis. Curr Rev Clin Exp Pharmacol 16: 330–340 33511945 10.2174/1574884716666210129100455

[emmm202013260-bib-0083] Roncero C , Valriberas‐Herrero I , Mezzatesta‐Gava M , Villegas JL , Aguilar L , Grau‐Lopez L (2020) Cannabis use during pregnancy and its relationship with fetal developmental outcomes and psychiatric disorders. A systematic review. Reprod Health 17: 25 32066469 10.1186/s12978-020-0880-9 PMC7027300

[emmm202013260-bib-0084] Rudd RA , Seth P , David F , Scholl L (2016) Increases in drug and opioid‐involved overdose deaths ‐ United States, 2010‐2015. MMWR Morb Mortal Wkly Rep 65: 1445–1452 28033313 10.15585/mmwr.mm655051e1

[emmm202013260-bib-0085] Sayed N , Liu C , Wu JC (2016) Translation of human‐induced pluripotent stem cells: from clinical trial in a dish to precision medicine. J Am Coll Cardiol 67: 2161–2176 27151349 10.1016/j.jacc.2016.01.083 PMC5086255

[emmm202013260-bib-0086] Seaton A , MacNee W , Donaldson K , Godden D (1995) Particulate air pollution and acute health effects. Lancet 345: 176–178 7741860 10.1016/s0140-6736(95)90173-6

[emmm202013260-bib-0087] Skolnick P (2018) The opioid epidemic: crisis and solutions. Annu Rev Pharmacol Toxicol 58: 143–159 28968188 10.1146/annurev-pharmtox-010617-052534

[emmm202013260-bib-0088] Stieb DM , Judek S , Burnett RT (2002) Meta‐analysis of time‐series studies of air pollution and mortality: effects of gases and particles and the influence of cause of death, age, and season. J Air Waste Manag Assoc 52: 470–484 12002192 10.1080/10473289.2002.10470794

[emmm202013260-bib-0089] Sun N , Yazawa M , Liu J , Han L , Sanchez‐Freire V , Abilez OJ , Navarrete EG , Hu S , Wang L , Lee A *et al* (2012) Patient‐specific induced pluripotent stem cells as a model for familial dilated cardiomyopathy. Sci Transl Med 4: 130ra147 10.1126/scitranslmed.3003552 PMC3657516 22517884

[emmm202013260-bib-0090] Takahashi K , Yamanaka S (2006) Induction of pluripotent stem cells from mouse embryonic and adult fibroblast cultures by defined factors. Cell 126: 663–676 16904174 10.1016/j.cell.2006.07.024

[emmm202013260-bib-0091] Takahashi K , Tanabe K , Ohnuki M , Narita M , Ichisaka T , Tomoda K , Yamanaka S (2007) Induction of pluripotent stem cells from adult human fibroblasts by defined factors. Cell 131: 861–872 18035408 10.1016/j.cell.2007.11.019

[emmm202013260-bib-0092] Templin C , Zweigerdt R , Schwanke K , Olmer R , Ghadri JR , Emmert MY , Muller E , Kuest SM , Cohrs S , Schibli R *et al* (2012) Transplantation and tracking of human‐induced pluripotent stem cells in a pig model of myocardial infarction: assessment of cell survival, engraftment, and distribution by hybrid single photon emission computed tomography/computed tomography of sodium iodide symporter transgene expression. Circulation 126: 430–439 22767659 10.1161/CIRCULATIONAHA.111.087684

[emmm202013260-bib-0093] Venkatesan P (2016) WHO report: air pollution is a major threat to health. Lancet Respir Med 4: 351 10.1016/S2213-2600(16)30014-5 27012457

[emmm202013260-bib-0094] Wang TW , Gentzke AS , Neff LJ , Glidden EV , Jamal A , Park‐Lee E , Ren C , Cullen KA , King BA , Hacker KA (2021) Characteristics of e‐cigarette use behaviors among US youth, 2020. JAMA Netw Open 4: e2111336 34097049 10.1001/jamanetworkopen.2021.11336 PMC8185598

[emmm202013260-bib-0095] Wei TT , Chandy M , Nishiga M , Zhang A , Kumar KK , Thomas D , Manhas A , Rhee S , Justesen JM , Chen IY *et al* (2022) Cannabinoid receptor 1 antagonist genistein attenuates marijuana‐induced vascular inflammation. Cell 185: e1623 10.1016/j.cell.2022.06.006 35750035

[emmm202013260-bib-0096] Winkler AS , Cherubini A , Rusconi F , Santo N , Madaschi L , Pistoni C , Moschetti G , Sarnicola ML , Crosti M , Rosso L *et al* (2022) Human airway organoids and microplastic fibers: a new exposure model for emerging contaminants. Environ Int 163: 107200 35349910 10.1016/j.envint.2022.107200

[emmm202013260-bib-0097] Wu JC , Rhee JW , Sallam K (2019) Electronic cigarettes: where there is smoke there is disease. J Am Coll Cardiol 74: 3121–3123 31856968 10.1016/j.jacc.2019.10.029 PMC10902211

[emmm202013260-bib-0098] Yu J , Vodyanik MA , Smuga‐Otto K , Antosiewicz‐Bourget J , Frane JL , Tian S , Nie J , Jonsdottir GA , Ruotti V , Stewart R *et al* (2007) Induced pluripotent stem cell lines derived from human somatic cells. Science 318: 1917–1920 18029452 10.1126/science.1151526

[emmm202013260-bib-0099] Zhao MT , Chen H , Liu Q , Shao NY , Sayed N , Wo HT , Zhang JZ , Ong SG , Liu C , Kim Y *et al* (2017) Molecular and functional resemblance of differentiated cells derived from isogenic human iPSCs and SCNT‐derived ESCs. Proc Natl Acad Sci U S A 114: E11111–E11120 29203658 10.1073/pnas.1708991114 PMC5748177

